# Expectations and satisfaction with antenatal care among pregnant women with a focus on vulnerable groups: a descriptive study in Ghent

**DOI:** 10.1186/s12905-015-0266-2

**Published:** 2015-12-02

**Authors:** Anna Galle, An-Sofie Van Parys, Kristien Roelens, Ines Keygnaert

**Affiliations:** Department of Obstetrics and Gynaecology, Faculty of Medicine and Health Sciences, International Centre for Reproductive Health, Ghent University, De Pintelaan 185, UZP 114, 9000 Ghent, Belgium

**Keywords:** Antenatal care, Expectations, Satisfaction, Vulnerable groups

## Abstract

**Background:**

Previous studies demonstrate that people’s satisfaction with healthcare influences their further use of that healthcare system. Satisfied patients are more likely to take part in the decision making process and to complete treatment. One of the important determinants of satisfaction is the fulfillment of expectations. This study aims to analyse both expectations and satisfaction with antenatal care among pregnant women, with a particular focus on vulnerable groups.

**Methods:**

A quantitative descriptive study was conducted in 155 women seeking antenatal care at the University Hospital of Ghent (Belgium), of whom 139 completed the questionnaire. The statistical program SPSS-21 was used for data analysis.

**Results:**

Women had high expectations relating to continuity of care and women-centered care, while expectations regarding availability of other services and complete care were low. We observed significantly lower expectations among women without higher education, with low income, younger than 26 years and women who reported intimate partner violence. General satisfaction with antenatal care was high. Women were satisfied with their relationship with the healthcare worker, however ; they evaluated the information received during the consultation and the organizational aspects of antenatal care as less satisfactory.

**Conclusions:**

In order to improve satisfaction with antenatal care, organizational aspects of antenatal care (e.g. reducing waiting times and increasing accessibility) need to be improved. In addition, women would appreciate a better provision of information during consultation. More research is needed for an in-depth understanding of the determinants of satisfaction and the relationship with low socio economic status (SES).

**Electronic supplementary material:**

The online version of this article (doi:10.1186/s12905-015-0266-2) contains supplementary material, which is available to authorized users.

## Background

Over the last two decades, increasing importance has been given to the opinions, expectations and experiences of women using health services, especially in the USA and Europe. Consumer satisfaction is playing an important role in quality of care reforms and health-care delivery [1, 2]. Patient satisfaction is a reflection of the patient’s judgment of different domains of health care, including technical, interpersonal, and organizational aspects [3]. International literature suggests that satisfaction with different aspects of received antenatal care improves health outcomes, continuity of care, adherence to treatment, and the relationship with the provider [3, 4]. The World Health Organization (WHO) recommends monitoring and evaluation of maternal satisfaction with public health care services, in order to improve the quality and efficiency of health care during pregnancy [1].

### Measurement of satisfaction

Quality of care is considered a multidimensional concept that has been given different meanings in the literature. Quality of care can be understood in light of two aspects: the resource structure of the care organization and patients’ preferences. Patient satisfaction has increasingly come to be used as an indicator of quality of care [5]. Patient satisfaction is a subjective and dynamic perception of the extent to which the patient’s expected health care needs are met [4]. The definition and conceptualization of satisfaction with health care is complex and multidimensional [2, 5]. To our knowledge there is no conceptual basis nor consistent measurement tool for satisfaction with antenatal care and a wide array of determinants seem to play a role. The existing studies demonstrate that factors such as waiting time before consultation, continuity in seeing the same health care worker, communication with the health care worker, setting and physical environment all impact on women’s satisfaction with antenatal care [4, 6]. More recently there is agreement that women’s satisfaction with antenatal care is determined by the interaction between their expectations and the characteristics of the healthcare they receive [3, 7]. In practice, expectations can refer to ideal health care, anticipated health care, or desired health care, and sometimes people do not have explicit expectations [8]. We use the second approach and define expectations as the pregnant women’s beliefs about the content, type and quality of care she will receive [7]. Christaens & Bracke [4] demonstrated the positive correlation of expectations and satisfaction, with fulfillment of expectations being one of the most consistent predictors of satisfaction.

### Vulnerable groups & health care satisfaction

Several observational studies demonstrate the association of late initiation of antenatal care and fewer antenatal visits (defined as ‘inadequate antenatal care’) with a number of socio-demographic factors in the USA and Europe [9–12]. These include young maternal age, migration background, low income, high parity, low level of education, low socio-economic status (SES), exposure to intimate partner violence (IPV), and not being in a steady relationship [10, 13–15]. Poor attendance at antenatal care is a well-known problem in vulnerable subgroups [15, 16]. Vulnerable populations can be defined as groups that face discrimination because of underlying differences in social status, which can lead to potential gaps in health or health care, considering race/ethnicity as well as other characteristics, such as disability and living conditions that pose special challenges to health care delivery (e.g., homeless, institutionalized, uninsured or homebound patients) [17, 18].

Increasing access to antenatal care for all women has become established as the key population-based public health intervention to address racial-ethnic disparities in perinatal outcomes [19, 20]. Adequate antenatal care by professional health care providers has been proven not only to reduce maternal, foetal and infant morbidity and mortality but also to result in improved maternal health status and parenting behaviours after the child is born [13]. Considering that dissatisfaction can be a major demotivating factor in the use of antenatal care facilities, enhancing satisfaction among vulnerable women can result in more regular consultations and a better relationship with the provider, eventually improving the quality of antenatal care [21]. At the same time we must recognize that many other factors such as social insurance, family support and transport play a role in patient health care use and outcomes in vulnerable groups, which will not be addressed by only improving patient satisfaction [17].

Across the continuum of antenatal, perinatal, and postnatal care, the assessment of maternal satisfaction with antenatal care is not well documented in Belgium. Christiaens & Bracke focused on the place of birth and maternal satisfaction, which gave valuable insights in this area, but specific research related to the satisfaction with antenatal care is lacking [22].

Hence, the general objective of this study was to assess expectations and satisfaction with antenatal care, with a focus on vulnerable women. Specific aims were to identify risk factors for low expectations and satisfaction and to explore which aspects of antenatal care could be improved in a hospital setting.

## Methods

A cross-sectional study was conducted at the antenatal clinic of Ghent University Hospital, Belgium. This antenatal clinic offers care for low- and high-risk pregnancies, the latter often referred from other hospitals. The standard antenatal visiting schedule at the clinic for a normal pregnancy consists of ten visits, which includes two consultations by a midwife and eight by a gynaecologist. In Belgium women are free to choose their own health care provider during pregnancy. Gynaecologists are principle health care providers for the vast majority of childbearing women in Belgium, while in many other parts of the world (e.g. Australia, the United Kingdom, Sweden and the Netherlands) midwives are the main providers [22–25]. In 2009 about 94.5 % of the pregnancies in Belgium were followed by an obstetrician/gynaecologist (OB/GYN) while 4.5 % were seen by a combination of a general practitioner and OB/GYN [10, 16].

### Recruitment

The study was approved by the Ethical Committee of the Ghent University Hospital (B670201419522) and all participants completed and signed an informed consent form. Between March and April 2014, pregnant women seeking antenatal care at the Ghent University Hospital were invited to participate in the study. Women were invited during the waiting time before the antenatal care consultation. Inclusion criteria were: being 18 years or older, having had at least one antenatal consultation in the current pregnancy, speaking Dutch or English, and being able to fill out an assisted questionnaire in Dutch or English. Women that did not meet the inclusion criteria or were not able to complete the questionnaire in private were excluded from the study. The study was limited to one questionnaire per woman and we did not impose limits on gestational age.

The researcher (a qualified midwife and Masters student in Health Promotion) invited the pregnant women to participate in the study while they were waiting for their antenatal consultation. The estimated number of women to be recruited was 100. This estimation was based on the number of women expected to attend the clinic and availability of the researcher (taken into consideration that only one woman at a time could fill in the questionnaire). In order to include women with low and high SES in the study, women were recruited during consultation hours of Kind & Gezin (Child & Family: A Flemish governmental institution offering psychosocial support during the perinatal period, with special attention to vulnerable groups) and during consultation hours of different OB/GYN with a substantial proportion of vulnerable pregnant women among their patient population.

The researcher introduced the study as a survey on satisfaction with antenatal care and briefly explained the procedure. Consenting women were handed an informed consent form, a participant information sheet and a questionnaire which was completed in a separate room. If the woman was unable to fill out the questionnaire in private (e.g. presence of family), she was excluded from the study. This was in order to avoid any influence by family/companions/spouse, etc. and to guarantee that all women followed the same procedure. To ensure that women with less developed literacy skills could also participate, the researcher was always present to clarify items or to answer questions, however keeping an appropriate distance (working on a computer). Only a small proportion of the women (ten women) needed assistance. Completing the questionnaire took an average of ten minutes. The questionnaire was anonymous, but the respondents had the option of providing their personal details if they were willing to be included in potential follow-up research. After completing the questionnaire the woman went directly to the health care provider or went back to the waiting room if the provider was not yet available.

### Questionnaires/measures

This cross-sectional study explored the expectations and satisfaction of pregnant women with antenatal care. Information on socio-demographic factors obtained from the questionnaire included: age, parity, number of living children, educational level completed, country of origin, income level, gestational age at the time of completing the questionnaire, number of antenatal visits, timing of first antenatal visit in the current pregnancy. A participant was classified as being of foreign descent if she or at least one of her parents was born outside the country of research. If both parents and the participant were born in Belgium, the participant was classified as having Belgian nationality. Two indicators, namely attained educational status and income level, were used to asses SES. Women with an academic degree (at college or university) were recoded as “higher education”, women without further education (no education, only primary or secondary school) were recoded as “without higher education”. Current household income of less than € 2000 per month was categorized as low income. Potential financial problems and financial dependency were further investigated by asking, “If you received an unexpected bill of 2824 euro how easy would it be for you to pay it within a week?”. Women indicating having no difficulties were recoded as “No” before the statistical analysis, while those indicating it would be “a little bit difficult” or “really difficult” were regarded as respectively experiencing moderate or serious financial distress. The questions regarding financial distress were based on the research of Wangel & Bidens Study Group [26] and the threshold of €2824 for financial distress in Belgium was based on the income distribution statistics of the European Commission [27]. Measurement of financial distress gives valuable information on a person’s financial situation and financial dependency. In addition, lifestyle factors were assessed including use of alcohol, drugs and medication. IPV prior to and during the current pregnancy was assessed using questions based on “The Abuse Assessment Screen” (AAS) [28]. The AAS is a questionnaire that asks about past and current emotional, sexual, and physical abuse, both prior to and during pregnancy. The AAS represents an important screening tool for obstetric populations and has mainly been tested with young and poor women [29]. We applied the short version of the AAS, which was previously used in a similar study conducted by one of the co-authors [30]. Assessment of vulnerability was done by identifying and analyzing a broad range of characteristics, according to our definition of vulnerability which takes into account all factors that can pose special challenges to health care delivery.

Satisfaction and expectations were measured by the PESPC (Patient Expectations and Satisfaction with Prenatal Care Instrument)-questionnaire, which was originally developed in the USA by Omar et al. [7]. The questionnaire was tested and validated by Omar et al. in a sample of women with low to middle socioeconomic status [7]. In 2013 test-retest analysis of the instrument was conducted by Prudencio et al. [31], with a positive correlation and strong magnitude (*r* = 0.82; *p* < 0.001) on the expectations domain and a positive correlation of moderate magnitude (*r* = 0.66; *p* < 0.001) for the satisfaction domain.

The original instrument was translated into Dutch and one of the 41 items was deleted. We decided to not include the item “I am satisfied with the services of a public health nurse as part of antenatal care” because these services do not exist in the Belgian health care context. Back translation methodology was followed with three independent translators, along with a test of the target language version with monolingual subjects [32]. In addition two experts established content validity.

The final instrument consisted of 40 items, divided into two domains: expectations and satisfaction. Each domain contained four subscales. The subscales in the expectations domain were: complete care, provider continuity, personalized care and availability of other services. The subscales in the satisfaction domain were: information, provider care, staff interest, and system characteristics. The questions to assess these subscales are listed in Additional files 1 and 2.

A Likert scale ranging from 1 (totally disagree) to 6 (totally agree) was used for evaluating the items, no neutral response option. The Likert scale was previously developed in that way by Omar et al. [7]. No items were reversed, high ratings on the scale corresponds with high expectations and high satisfaction. Construct validity of the PESPC’s final instrument was verified through exploratory factor analysis with Varimax rotation for the two domains, expectations and satisfaction. In both domains, all the factor loads were grouped and were above 0.30. The highest communality value in the expectations domain was identified for item 9 (0.77) and the lowest for item 1 (0.35), in the satisfaction domain the highest communality value was identified for item item 8 (0.79) and the lowest for item 19 (0.34). The final instrument had a good internal consistency, with a Cronbach’s α value of 0.70 for expectations and 0.82 for satisfaction. The complete questionnaire can be found in Additional file 3.

### Data-analysis

The statistical program SPSS-21 was used for data analysis. Descriptive analysis was performed for all the variables. We analysed nominal and categorical variables using Pearson’s chi-squared and Fisher’s exact test for differences between two groups. Groups were created based on the absence or presence of specific characteristics (of vulnerability). For tables with expected count less than five the Fisher Exact test was used. Each variable was analysed separate without controlling for confounding factors.

To analyse differences between groups regarding satisfaction and expectations (continuous variables) the Independent Sample T-test was performed and the *p*-value was computed. The level of significance was fixed at 0.05. Normality was determined graphically by using a Q-Q plot. The assumption of homogeneity of variance was tested by using the Levene's Test of Equality of Variances. This research adhered to the STROBE guidelines for cross-sectional studies [33].

## Results

**Fig. 1:**
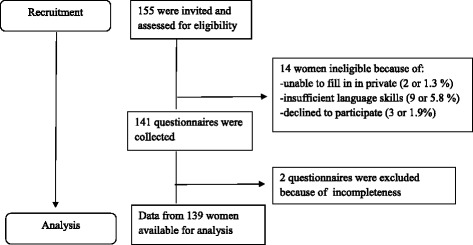
Flow diagram recruitment

Figure 1 shows that of the 155 women who were eligible from the sample, 141 were recruited (participation rate of 90.67 %) and 139 women completed the full questionnaire (response rate of 89.68 %) [34]. The main reason for exclusion was insufficient language skills, only three women declined participation. Most women chose to fill out the questionnaire in Dutch (95 %) and 5 % in English.

The average age of the respondents was 30.5 ± 4.6 years, range 18–40 years, and the vast majority (94 %) were cohabiting or married. As for educational status: 69.1 % had a higher education qualification (university or college), 20.1 % had a secondary education and 10.8 % did not complete secondary school. About three quarters (72.7 % or *n* = 101) of the women were of Belgian nationality. With regard to income we found that 10 % of the women had an income lower than the national minimum wage (less than €800 or €1000, depending on marital status). More than a quarter of the women reported serious financial distress. A third (30.2 % or *n* = 42) of the women were primigravida and 15.1 % (*n* = 21) of the pregnancies were unplanned. The first antenatal care visit was later than 12 weeks of gestation for 5 % (*n* = 7) of the women. The average timing of the first appointment was at seven weeks, ranging between the second week (medically assisted conception) and the 25th week. The main healthcare provider in the antenatal period was the OB/GYN, with the number of consultations varying between 2 and 20 at the time of completing the questionnaire. The average number of consultations with the OB/GYN was seven and the average number of consultations with the midwife was two. Gestational age at the time of completing the questionnaire varied between 7 and 40 weeks with an average of 29 weeks. More details are presented in Table 1.

**Table 1:** Descriptive Statistics: Socio-demographic data

Age	
18–25	12 % (*n* = 17)
26–30	40 % (*n* = 55)
31–35	32 % (*n* = 45)
>35	16 % (*n* = 22)
Origin	
Belgian	73 % (*n* = 101)
Foreign Descent	27 % (*n* = 38)
Education (*n* = 139)	
No education or primary	11 % (*n* = 15)
Secondary school (*n* = 28)	20 % (*n* = 28)
Higher Education (*n* = 96)	69 % (*n* = 96)
Household income (*n* = 139)	
< € 800	3 % (*n* = 4)
Between € 800 and € 1000	9 % (*n* = 13)
Between € 1000 and € 1500	8 % (*n* = 11)
Between € 1500 and € 2000	8 % (*n* = 11)
> € 2000	72 % (*n* = 100)
Financial distress (*n* = 139)	
No	47 % (*n* = 65)
Moderate	27 % (*n* = 38)
Serious	26 % (*n* = 36)
Marital status (*n* = 139)	
Married/cohabiting	94 % (*n* = 131)
Divorced	1 % (*n* = 2)
Single	4 % (*n* = 6)
Pregnancy	
Planned	85 % (*n* = 118)
Unplanned	15 % (*n* = 21)
First Consultation	
Before 12 weeks gestation	95 % (*n* = 132)
After 12 weeks gestation	5 % (*n* = 7)
Gravidity	
Primigravida	70 % (*n* = 96)
Multigravida	30 % (*n* = 42)
Gestational Age	
First Trimester	7 % (*n* = 9)
Second Trimester	22 % (*n* = 26)
Third Trimester	71 % (*n* = 86)

One third (33.8 %) of the women used substances during pregnancy: 1.4 % used medication (for example tranquilizers, sleeping pills, anti-anxiety drugs or anti-depressants), 1.4 % used illicit drugs (for example cannabis, amphetamines, ecstasy or cocaine), 20.8 % used alcohol and 13 % smoked cigarettes, more details are presented in Table 2.

**Table 2:** Descriptive Statisitics: use of substances

Use of alcohol (*n* = 139)		Use of medication (*n* = 139)	
Never	26.6 % (*n* = 37)	Never	79.1 % (*n* = 110)
Not during pregnancy	52.5 % (*n* = 73)	Yes, but not in the 12 months prior to the current pregnancy	16.5 % (*n* = 23)
< Once a month during pregnancy	13.7 % (*n* = 19)	Yes, in the 12 months prior to the current pregnancy	2.9 % (*n* = 4)
One to three times a month during pregnancy	5.0 % (*n* = 7)	During pregnancy	1.4 % (*n* = 2)
One to three times a week during pregnancy	1.4 % (*n* = 2)		
(Almost) every day during pregnancy	0.7 % (*n* = 1)		
Smoking (*n* = 139)		Drug-use (cannabis, amphetamines, ecstasy, cocaine) (*n* = 139)	
Never	87.1 % (*n* = 121)	Never	79.9 % (*n* = 111)
Almost never (less than one cigarette a week, but not daily) during pregnancy	2.2 % (*n* = 3)	Yes, but not in the 12 months prior to the current pregnancy	13.7 % (*n* = 19)
Now and again (at least one cigarette a week) during pregnancy	2.9 % (*n* = 4)	Yes, in the 12 months prior to the current pregnancy	5.0 % (*n* = 7)
Daily during pregnancy	7.9 % (*n* = 11)	During pregnancy	1.4 % (*n* = 2)

With regard to IPV, 5.8 % (*n* = 8) of the women reported to have been physically abused in the 12 months prior to or during pregnancy, 1.4 % (*n* = 2) reported sexual abuse and 4.2 % (*n* = 6) emotional abuse. The detailed IPV prevalence rates of our study population are presented in Table 3.

**Table 3:** Descriptive Statistics: Intimate Partner Violence

	Physical violence (*n* = 139)	Emotional violence (*n* = 139)	Sexual violence (*n* = 139)	At least one type of violence (physical/emotional/sexual) (*n* = 139)
No	94.2 % (*n* = 131)	95.7 % (*n* = 133)	98.6 % (*n* = 137)	92.8 % (*n* = 129)
Yes, 12 months before pregnancy	2.9 % (*n* = 4)	0.7 % (*n* = 1)	0.7 % (*n* = 1)	2.9 % (*n* = 4)
Yes, during pregnancy	2.9 % (*n* = 4)	3.6 % (*n* = 5)	0.7 % (*n* = 1)	4.3 % (*n* = 6)

### Expectations and satisfaction related to different aspects of antenatal care

The average score among all women in our study was calculated for expectations and satisfaction and possible intervals and averages were calculated for each subscale (see Table 4). Calculations were made according to the scoring procedure used by Prudencio et al. [31].

**Table 4:** Descriptive Statistics scales & subscales: Expectations & Satisfaction

Scales & Subscales	Number of items	Score possible interval	Score average (SD)	Score percentage
Expectations (*n* = 133)	12	12–72	38.40 (11.69)	53.3 %
Complete Care (*n* = 136)	4	4–24	10.90 (4.64)	45.4 %
Provider Continuïty (*n* = 136)	2	2–12	7.03 (3.21)	58.6 %
Personalized Care (*n* = 136)	4	4–24	16.18 (5.36)	67.4 %
Other Services (*n* = 138)	2	2–12	4.52 (2.41)	37.7 %
Satisfaction (*n* = 117)	28	28–168	136.86 (15.70)	81.5 %
Information (*n* = 133)	6	6–36	28.33 (4.27)	78.7 %
Provider Care (*n* = 135)	6	6–36	31.26 (3.82)	86.3 %
Staff Interest (*n* = 136)	6	6–36	30.54 (3.89)	84.8 %
System Characteristics (*n* = 127)	10	10–60	46.83 (6.77)	78.0 %

Expectations about different aspects of antenatal care were assessed by the subscales (complete care, provider continuity, personalized care, other services). Percentages of less than 50 % demonstrate that women slightly disagree with the statements and indicate low expectations. The highest average score was obtained for personalized care with 67.4 % and the lowest average score was obtained for other services with 37.7 %. The subscales complete care and personalized care showed an average score of 45.4 and 67.4 % respectively (more details are represented in Table 4).

In the satisfaction scale the highest scores were obtained for the subscales ‘provider care’ and ‘staff interest’, 86.3 and 84.8 % respectively. The lowest scores were observed for the subscales ‘information’ and ‘system characteristics’, 78.7 and 78.0 % respectively. More details are available in Table 4 & Additional file 2.

### Risk factors for low expectations and/or satisfaction with antenatal care

Comparing average expectations sum scores by means of an independent T-test, we found the following variables to be significant: educational level, income, age, marital status and reporting IPV. We observed significantly lower expectations among women without higher education, with low income, younger than 26 years, single or divorced women and women who reported IPV. Origin showed a trend towards significance (*P* < 0.1), women with a foreign descent seem to have lower expectations. In the satisfaction domain, no variables were statistically significant. We present more details in Table 5.

**Table 5:** Independent Sample T-test: Expectations & Satisfaction: Averages (DS) (**P < 0.05 *P < 0.1)

Expectations	Average (SD)	t (Df)-value *P*-Value
Educational Level**		
No higher Education (*n* = 40)	34.07 (14.71)	t (53.94) = −2.442 *P* 0.018
Higher education (*n* = 93)	40.26 (9.63)	
Origin*		
Belgian (*n* = 100)	39.76 (9.10)	t (38.30) = 1.79 *P* 0.082
Foreign Descent (*n* = 33)	24.27 (16.87)	
Household-income**		
Less than 2000 euro (*n* = 36)	33.42 (15.99)	t (43.61) = −2.42*P* 0.020
More than 2000 euro (*n* = 97)	40.25 (9.06)	
Marital Status**		
Divorced, single (*n* = 6)	32.86 (15.75)	t (131) = −3.067 *P* 0.003
Co-habiting, married (*n* = 113)	38.71 (11.43)	
Age**		
18–25 years (*n* = 17)	31.00 (13.27)	t (19.41) = 2.511 *P* 0.021
26–40 years (*n* = 116)	39.48 (11.10)	
Reporting IPV**		
Yes (*n* = 10)	31.00 (11.53)	t (131) = 2.108 *P* 0.037
No (*n* = 123)	39.00 (11.73)	
Late initiation PNC		
Yes (*n* = 7)	37.57 (15.60)	t (131) = 0.192 *P* = 0.848
No (*n* = 126 )	38.44 (11.52)	
Satisfaction	Average (SD)	t (Df)-value *P*-value
Educational Level		
No higher Education (*n* = 40)	139.75 (17.84)	t (117) = 0.059 *P* = 0.953
Higher education (*n* = 93)	135.60 (14.61)	
Origin		
Belgian (*n* = 100)	135.96 (14.51)	t (117) = −0.105 *P* = 0.272
Foreign descent (*n* = 38)	139.66 (18.94)	
Household-income		
Less than 2000 euro (*n* = 36)	139.68 (18.68)	t (47.28) = 1.079 *P* = 0.286
More than 2000 euro (*n* = 97)	135.78 (13.37)	
Marital Status		
Divorced, single (*n* = 6)	137.67 (21.55)	t (117) = 0.921 *P* = 0.359
Co-habiting, married (*n* = 113)	136.81 (15.46)	
Age		
18–25 years (*n* = 17)	140.93 (19.60)	t (117) = −1.033 *P* = 0.304
26–40 years (*n* = 116)	136.66 (15.14)	
Reporting IPV		
Yes (*n* = 10)	132.50 (20.70)	t (117) = 0.696 *P* = 0.488
No (*n* = 123)	137.09 (15.48)	
Late initiation PNC		
Yes (*n* = 7)	131.50 (21.10)	t (117) = 0.857 *P* = 0.393
No (*n* = 126 )	137.14 (15.43)	

### Risk factors (financial distress & IPV & unplanned pregnancy) for inadequate antenatal care

Table 6 illustrates the relationship between financial distress, unplanned pregnancy, IPV and several socio-demographic factors. Women without higher education (*P* < 0.001), younger than 26 years old (*P* < 0.001), with low income, divorced or single women (*P* = 0.004) and women who smoked (*P* = 0.020) reported significantly higher levels of financial distress during pregnancy. A significant association (*P* < 0.001) was also found between financial stress and being of foreign descent. In our study only 16.8 % of women with Belgian nationality reported financial distress compared to 50 % of women of foreign descent.

**Table 6:** Association of different risk factors (Chi Square test & Fisher Exact test)

	Percentage of financial stress	*P*-value	Percentage of IPV	*P*-value	Unplanned pregnancy	*P*-value
Age						
18–25 (*n* = 17)	70.6 % (*n* = 12)	*P* < 0.001	29.4 % (*n* = 5)	*P* = 0.003	41.2 % (*n* = 7)	*P* = 0.001
26–40 (*n* = 122)	19.7 % (*n* = 24)		4.1 % (*n* = 5)		11.5 % (*n* = 14)	
Marital Status						
Married/Cohabiting (*n* = 131)	22.9 % (*n* = 30)	*P* = 0.004	20.9 % (*n* = 9)	*P* < 0.001	13.0 % (*n* = 17)	*P* = 0.018
Divorced/Single (*n* = 8)	75.0 % (*n* = 6)		1.0 % (*n* = 1)		50.0 % (*n* = 4)	
Education						
Without Higher Education (*n* = 43)	55.8 % (*n* = 24)	*P* < 0.001	20.9 % (*n* = 9)	*P* < 0.001	37.2 % (*n* = 16)	*P* < 0.001
Higher Education (*n* = 96)	12.5 % (*n* = 12)		1.0 % (*n* = 1)		5.2 % (*n* = 5)	
Origin						
Foreign Descent (*n* = 38)	50 % (*n* = 19)	*P* < 0.001	10.5 % (*n* = 4)	*P* = 0.461	36.8 % (*n* = 14)	*P* < 0.001
Belgian (*n* = 101)	16.8 % (*n* = 17)		5.9 % (*n* = 6)		6.9 % (*n* = 7)	
Income						
Less than 2000 euro (*n* = 39)	66.7 % (*n* = 26)	*P* < 0.001	15.4 % (*n* = 6)	*P* = 0.029	38.5 % (*n* = 15)	*P* < 0.001
More than 2000 euro (*n* = 100)	10.0 % (*n* = 10)		4.0 % (*n* = 4)		6.0 % (*n* = 6)	
Smoking during pregnancy						
Smoking (*n* = 18)	50.0 % (*n* = 9)	*P* = 0.020	30 % (*n* = 3)	*P* = 0.122	33.3 % (*n* = 6)	*P* = 0.032
Not smoking (*n* = 121)	22.3 % (*n* = 27)		5.8 % (*n* = 7)		12.4 % (*n* = 15)	

Furthermore, a significant association was found between IPV and other risk factors for inadequate antenatal care (see Table 6). Women without higher education (*P* < 0.001), younger than 26 years old, with low income (*P* < 0.001) and divorced or single women (*P* < 0.001) reported significantly more IPV in the 12 months prior to or during pregnancy. Only 4 % of women with an income of more than 2000 euro reported IPV compared to15.4 % of women with an income of less than 2000 euro.

We found significantly higher percentages of unplanned pregnancy in women of foreign descent (*P* < 0.001), without higher education (*P* < 0.001), younger than 26 years (*P* = 0.001), with low income (*P* < 0.001), divorced or single women (*P* = 0.018) and women who smoked during pregnancy (*P* = 0.032).

## Discussion

We know that increasing the satisfaction of pregnant women with antenatal services can result in better health outcomes for mother and child. This study analysed both expectations and satisfaction with antenatal care.

### Satisfaction with antenatal care

In our study satisfaction scores where higher than those previously reported (with the same scale) in the United States, which may indicate that in Belgium women are very satisfied with antenatal care [7]. In the literature we found similar results indicating that overall satisfaction with the health care system in Belgium is very high [1, 22]. Bleich, Özaltin & Murray examined satisfaction with health systems in 21 European Union countries and Belgium was ranked second after Austria [1]. Also Christiaens & Bracke found high satisfaction rates among women attending the Flemish perinatal healthcare system compared to women in the Netherlands [22].

### Different aspects of antenatal care

For the subscales regarding the relationship with the healthcare provider (‘staff interest’ and ‘provider care’) we observed high satisfaction levels. The subscale ‘information’ had lower scores. Research suggests that more efforts should be made to improve the transfer of essential information during antenatal consultations: women seem to be satisfied with technical aspects of antenatal care but also report a lack of communication by health care professionals [3, 6, 35–37]. The OB/GYN is mainly addresses medical issues and time is limited. A recent study [38] has shown that women who have a midwife as their antenatal health care provider report fewer communication problems than women who receive care from other types of clinicians. This suggests that the assignment of a midwifery led care option for low-risk pregnancies may result in better communication with the health care provider during pregnancy, as may the introduction of more consultations with a midwife in the normal antenatal care trajectory in Belgium. In many other countries midwives are the main health care providers during pregnancy. Midwifery led care is a model which has demonstrated effectiveness, satisfaction, and lower costs in several studies; the benefits of introducing this model in Belgium should be further explored [38, 39].

The subscale ‘system characteristics’ had the lowest scores and in particular the item ‘waiting times’ (see Additional file 2). This is in line with previous research addressing barriers to antenatal care [3, 40, 41]. Sunil et al. [41] reported service related barriers to be the most significant factor influencing the decision when to start antenatal care. Service barriers included: not having child care or transportation, having to wait too long to get an appointment, and having to wait too long in the waiting room to see the doctor or nurse. In order to improve antenatal care policy makers and providers should focus more on features of the antenatal care setting (such as accessibly, waiting times and availability of ancillary services) instead of further medicalization of pregnancy and concentrating mainly on technical proficiency [40].

### Factors significantly associated with low expectations

Many studies attempt to identify how various factors directly influence patient perceptions (or more typically patient satisfaction) and patient expectations have rarely been explicitly studied. This makes it difficult, if not impossible, to determine whether differences in satisfaction reflect expectations, perceptions, definitions or criteria, or experiences [42]. Our results revealed different risk factors for low expectations of antenatal care. Women with low income, without higher education, <26 years old, of foreign descent (significant trend), single or divorced women and women who reported IPV had significantly lower expectations of antenatal care. This can be explained by various factors that influence patients’ expectations: the patient’s previous experiences, social and cultural norms (e.g. those with greater education or authority are more critically), patient demographics (e.g. age, gender), and last, but certainly not least, the extent to which the patient has knowledge of what s/he should expect [42]. Our findings were in line with previous research, which has found that women with low SES or women who are less familiar with the health care system (such as women with foreign descent) have lower expectations regarding antenatal care [31, 42].

### Factors significantly associated with low satisfaction

No significant relationship was found between satisfaction and SES in our study and other risk factors for low satisfaction with antenatal care could not be identified. Income and education were used as indicators for SES, while ethnicity was not included as a socio-economic indicator in our study. Consensus about the influence of SES on satisfaction is lacking in the literature. It remains unclear if women with low SES are less or more satisfied with antenatal care. Prudencio et al. [31] used the same instrument to measure satisfaction and only found a significant relationship between marital status and satisfaction, while income and education were not found to be related to satisfaction. Some studies have suggested that antenatal care can be particularly frustrating for women with low SES, due to experiences such as discrimination or stereotyping [36, 41, 43]. Language barriers and medical jargon may impede communication for women with lower literacy levels and migrants [36, 44]. On the other hand many studies indicate positive experiences of women with low SES [1, 31]. Bleich et al. observed a weak but statistically significant association between education and satisfaction with health care; people with some college education were less likely to be satisfied with the health system compared to people without a high school qualification [1].

The theory of Fishbein & Ajzen [45] can partially explain the higher satisfaction of women with low SES. Women with low SES have lower expectations about the care they will receive. These lower expectations are easier to fulfill and as a consequence women are more satisfied with the care they receive. With increasing levels of education, women′s expectations increases, which may explain why high educated women tend to be less satisfied. Our results also seem to support this hypothesis since women with low SES had significantly lower expectations than women with high SES. Women without higher education and low income women had higher average satisfaction scores, but these differences were not significant. Vulnerable women do not seem less satisfied but many other factors may impede their access to antenatal care. In our study several associations between factors of vulnerability were demonstrated. We found an association between financial stress and being of foreign descent. Literature has shown that migrants pay fewer and later antenatal visits, have poorer pregnancy outcomes and are more at risk of unintended pregnancies [46, 47]. Financial barriers are still a main cause of not seeking antenatal care for undocumented migrants in Belgium. Antenatal care is considered as urgent medical care which means that the social welfare system (OCMW) should reimburse these medical costs for those who are undocumented. However, the implementation and interpretation of this regulation varies from doctor to doctor and from OCMW to OCMW, creating ambiguity and discrimination [48]. If policymakers want to guarantee universal access to antenatal care, removing organizational barriers should be a higher priority as well as removing financial barriers.

Finally, our study equally also confirmed the association of IPV with other risk factors for inadequate antenatal care (low SES, unplanned pregnancy, financial distress, and <26 years old). Over the past decades, research has generated growing evidence that IPV is a prevalent problem that is linked to a broad range of adverse health outcomes and risk behaviour [14, 49]. Antenatal home visiting programs and some multifaceted counselling interventions produced promising results to tackle IPV and other risk factors simultaneously in vulnerable pregnant women [49, 50]. Currently, psychosocial services for vulnerable women in Belgium, provided by Kind & Gezin (K&G), are mainly directed towards the postnatal period. K&G offers free home visits by a district nurse for every mother in the first few months after birth. K&G could play an important role in testing out and introducing specific antenatal care programs for vulnerable women in Belgium.

We can conclude that vulnerable women are not less satisfied about the care they receive and many other factors besides their experience as a patient, such as social network, insurance, and health literacy, play a role in antenatal care attendance. Therefore improving satisfaction among vulnerable women may be of limited use for increasing antenatal care attendance, instead a wide array of determinants should be tackled simultaneously to increase access for vulnerable groups.

### Limitations

The study has some limitations. Using a face to face recruitment and data collection procedure, we were able to obtain high response rate of 89.7 %. On the other hand personal and direct contact is a well-known risk for response bias, although no health care workers of the antenatal consultation were involved in the recruitment procedure [34]. Despite the high response rate, some selection bias could not be avoided as obviously women without any access to antenatal care were not reached. Also women younger than 18 years old were excluded from the study for ethical reasons, they only can be interviewed with parental permission. Qualitative research using snowball sampling could be a better approach for reaching these groups.

The issue of acquiescence response bias needs consideration for the questions concerning satisfaction: this section was the last part of the questionnaire and mainly high scores and little variation were obtained. Another limitation that should be taken into consideration is the wide range of gestational age, women at 7 weeks gestational age (with at least one previous antenatal care consultation) may have little experience with antenatal care compared to women at the end of their pregnancy.

Although previous research supported the validity and reliability of the questionnaire, it should be tested in more demographically and culturally diverse samples. Satisfaction with health care is a multidimensional construct and hard to measure [25]. We cannot be sure that the attributes chosen in the scales are those most important to quality of care. It is common that general questions are given high rates [51]. In order to have a more complete understanding of women’s expectations and satisfaction with antenatal care, specific questions about the importance of certain aspects of care could be added to the questionnaire [52]. Further research with a greater sample size is recommended to broaden the in-depth understanding of the determinants of satisfaction with the healthcare system and the relationship with SES. Future research can also address more diverse health care settings.

## Conclusion & implications for practice

We do believe that this study provides useful insights for enhancing care for pregnant women in the antenatal period. The association between several risk factors (including smoking & unplanned pregnancy, foreign descent & financial distress, IPV & low SES) of vulnerability was demonstrated in our study. Furthermore we were able to demonstrate the link between some socio-demographic characteristics and low expectations about antenatal care. Antenatal care can be a window of opportunity to address these risk factors simultaneously, as they are closely linked to each other, and affect the health of both child and mother .

Our study highlighted the importance of the organizational aspects of care and the need for more information during consultations in order to achieve greater maternal satisfaction with antenatal health care. Institutions offering antenatal care should consider practical arrangements to remove some of the organizational barriers that affect the satisfaction of women including reduction of waiting times, improvement of transportation facilities and provision of walk-in consultations. A relatively small investment could have a great impact on the satisfaction of women, which can improve maternal and newborn health. Midwives can play an important role in improving the provision of adequate information and health promotion during pregnancy.

## Additional files

Additional file 1:
**Scale Expectations.** An overview of the items used to assess the subscales in the expectations domain. (PDF 143 kb) Additional file 2:
**Scale Satisfaction.** An overview of the items used to assess the subscales in the satisfaction domain. (PDF 148 kb) Additional file 3:
**Questionnaire.** The complete questionnaire used in this study, a version in English and Dutch respectively. (PDF 426 kb)

## Abbreviations

ANC: Antenatal Care
ICRH: International Centre for Reproductive Health
IPV: Intimate Partner Violence
K&G: Kind & Gezin
OB/GYN: Obstetrician/ Gynaecologist
OCMW: Openbaar Centrum voor Maatschappelijk Welzijn
PESPC: Patient Expectations and Satisfaction with Prenatal Care Instrument
SES: Socio Economic Status
SPSS: Statistical Package for Social Sciences
USA: United States of America
WHO: World Health Organization
