# Supplementary material for: Expectations and satisfaction with antenatal care among pregnant women with a focus on vulnerable groups: a descriptive study in Ghent
Source: BMC Womens Health. 2015 Dec 2;15:112. doi: 10.1186/s12905-015-0266-2 (PMC4667492; doi:10.1186/s12905-015-0266-2)
Supplement: Additional file 1: — Scale Expectations. An overview of the items used to assess the subscales in the expectations domain. (PDF 143 kb) [file 12905_2015_266_MOESM1_ESM.pdf]

Annex 1: Scale Expectations

Complete Care
I expected to be seen sooner for my first antenatal visit
I expected to have my antenatal visits take a long time
I expected to get more from my antenatal visits than being weighed and having my baby's heart checked
I expected to receive information during my visits without having to ask so many questions
Provider Continuity
I expected to have one provider (=gynaecologist, midwife or doctor) that I routinely see for my antenatal visits
I expected to have the provider that I routinely see deliver my baby
Personalized Care
I expected my provider to care how I feel mentally as well as physically
I expected my provider to be gentle during my physical exam
I expected someone to listen to my problems
I expected a referral when I tell the clinic/office staff about a problem
Other Services
I expected the services of a social worker to be part of antenatal care
I expected the services of a nutritionist to be part of antenatal care
