# Supplementary material for: Expectations and satisfaction with antenatal care among pregnant women with a focus on vulnerable groups: a descriptive study in Ghent
Source: BMC Womens Health. 2015 Dec 2;15:112. doi: 10.1186/s12905-015-0266-2 (PMC4667492; doi:10.1186/s12905-015-0266-2)
Supplement: Additional file 2: — Scale Satisfaction. An overview of the items used to assess the subscales in the satisfaction domain. (PDF 148 kb) [file 12905_2015_266_MOESM2_ESM.pdf]

Annex 2: Scale Satisfaction

Information
I am satisfied with the explanation my provider gave to me of what was going to happen during my antenatal visits
I am satisfied with the explanation my provider gave to me about medical procedures
I am satisfied with the information my provider gave to me about how things are going with my pregnancy
I am satisfied with the kinds of things my provider discussed during my antenatal visits
I am satisfied with the explanation my provider gave to me about what I can expect about parenting a new-born
I am satisfied with the way my provider has prepared me for labour and delivery
Provider Care
I am satisfied with the way my provider treats me
I am satisfied with the respect that I am shown by my provider
I am satisfied with the quality of care that I receive from my provider
I am satisfied with the way I am made to feel that I am not wasting my provider's time
I am satisfied with being able to ask questions without embarrassment
I am satisfied with not having to repeat my story every time I come in for a visit
Staff Interest
I am satisfied with the way the staff expresses concern about my overall personal situation
I am satisfied with the time the staff spends talking about things of interest to me
I am satisfied with the way the staff treats me
I am satisfied with the time the staff takes with me even though I do not have problems with this pregnancy
I am satisfied with the interest and concern the staff has shown me
I am satisfied with the way the staff deals with all my medical problems
System Characteristics
I am satisfied with the amount of time I wait to be seen by my provider
I am satisfied with the total amount of time I spend at the office/clinic
I am satisfied with the parking facilities of the office/clinic
I am satisfied with the waiting room facilities of the office/clinic
I am satisfied with the examination room of the office/clinic
I am satisfied with my ability to schedule antenatal visits at a time convenient for me
I am satisfied with how easy it is to reschedule my antenatal visits
I am satisfied with how easy it was to get antenatal care early in my pregnancy (that is, before the fourth month)
I am satisfied with having all the recommended tests
I am satisfied with the number of antenatal visits I made until now
