# Supplementary material for: Expectations and satisfaction with antenatal care among pregnant women with a focus on vulnerable groups: a descriptive study in Ghent
Source: BMC Womens Health. 2015 Dec 2;15:112. doi: 10.1186/s12905-015-0266-2 (PMC4667492; doi:10.1186/s12905-015-0266-2)
Supplement: Additional file 3: — Questionnaire. The complete questionnaire used in this study, a version in English and Dutch respectively. (PDF 426 kb) [file 12905_2015_266_MOESM3_ESM.pdf]

Annex 3: Questionnaire (English and Dutch version)

UNIVERSITEIT GENT

Instructions for completing the questionnaire

Dear Miss,

My name is Anna Galle, I am a midwife and a student Master in Health Promotion at the University of Ghent. I am writing a thesis dealing with the barriers to prenatal care in Flanders. For this, I am looking for pregnant women who are willing to complete a questionnaire about how satisfied they are about prenatal care. Completing the questionnaire takes about 15 minutes of your time, I will be present and assist if necessary. On page 8 & 9 you will find more information about the study and the informed consent form.

The questionnaire consists of two parts, one part with general questions and one part with statements. This study is an independent study, only researchers associated with this study will have access to the data. All information from this questionnaire will be treated as strictly confidential.

QUESTIONNAIRE: GENERAL QUESTIONS

1. Date at which this questionnaire was completed: ……….. / ………….. / 2014

2. What is your date of birth? ………… / ……….. / 19…………

3. What is your nationality?

   ……..

4. What is the nationality of your parents?

   Father: ……….

   Mother: …….

5. How many weeks are you pregnant at this moment? ….. weeks

6. How many times have you been pregnant (miscarriages, abortuses, stillbirths and current pregnancy included) ?

….. pregnancies

7. How many children alive do you have now? ….. children

8. Age of the children:

Child 1: …………….years old Child 2: ……………. years old

Child 3: …………….years old Child 4: …………….. years old

Child 5: …………….years old Child 6: …………….. years old

9. How many miscarriages (before the 20th week of pregnancy) have you had?

….. miscarriages

10. Was this pregnancy planned?
    - o Yes
    - o No

11. How many weeks were you pregnant (current pregnancy) when you had your first appointment with a midwife, doctor or gynaecologist?

….. weeks

12. How many prenatal visits did you have with a midwife during your current pregnancy? ……

13. How many prenatal visits did you have with a gynaecologist during your current pregnancy? …..

14. How many prenatal visits did you have with a family doctor during your current pregnancy? …..

15. What is the highest level of education you have completed?
    - Non or primary
    - Secondary high school
    - Higher education
16. What is your current marital status?
    - Married/cohabiting
    - Divorced
    - Lat relationship
    - Single
17. How much is your current household income?
    - Less than 800 euro
    - Between 800 and 1000 euro
    - Between 1000 and 1500 euro
    - Between 1500 and 2000 euro
    - More than 2000 euro
18. If you had to pay an unexpected bill of 2222 euro, how easy would it be to pay it within one week?
    - No problem
    - A little bit difficult
    - Really difficult
19. Did you ever smoke?
    - No, I never smoked
    - Yes
20. Are you currently smoking?
    - Yes, at least one cigarette a day
    - Yes, now and again (at least one cigarette a week, but not daily)
    - Almost never (less than one cigarette a week)
    - Not

21. How often do you consume alcohol?

    - Never
    - Not during pregnancy
    - Occasional, less than once a month
    - One to three times a month
    - One to two times a week
    - (almost) every day

22. Did you ever take sleeping pills, tranquilizers, anti-anxiety drugs, anti-depressants or other drugs for psychological problems?

    - Never
    - Yes, but not in the 12 months prior to the current pregnancy
    - Yes, in the 12 months prior to the current pregnancy
    - Yes, during this pregnancy

23. Did you ever use drugs such as cannabis, amphetamines, ecstasy or cocaine?

    - Never
    - Yes, but not in the 12 months prior to the current pregnancy
    - Yes, in the 12 months prior to the current pregnancy
    - Yes, during this pregnancy

24. Did your (ex-) partner hit you, slapped you, kicked you or otherwise physically hurt you?

    - Yes, in the 12 months prior to the current pregnancy
    - Yes, during this pregnancy
    - No

25. Did your (ex-) partner touched you in a sexual way when you didn't want to or forced you to have sexual activities?

    - Yes, in the 12 months prior to the current pregnancy
    - Yes, during this pregnancy
    - No

26. Did your (ex-) partner belittled or humiliated you in front of other people, did things do intimidate or scare you on purpose or did other thing to hurt you emotionally?

    - Yes, in the 12 months prior to the current pregnancy
    - Yes, during this pregnancy
    - No

Please indicate your state of agreement on a scale of 1 to 6, from “I disagree strongly” to “I agree strongly”. Try to remember what expectations you had at the beginning of your pregnancy about prenatal care when you evaluate the statements.”.

YOUR EXPECTATIONS AT THE BEGINNING OF THE PREGNANCY

	I disagree strongly	I disagree moderately	I disagree slightly	I agree slightly	I agree moderately	I agree strongly
I expected to be seen sooner for my first prenatal visit	1	2	3	4	5	6
I expected to have my prenatal visits take a long time	1	2	3	4	5	6
I expected to get more from my prenatal visits than being weighed and having my baby's heart checked	1	2	3	4	5	6
I expected to receive information during my visits without having to ask so many questions	1	2	3	4	5	6
I expected to have one provider (=gynaecologist, midwife or doctor) that I routinely see for my prenatal visits	1	2	3	4	5	6
I expected to have the provider that I routinely see deliver my baby	1	2	3	4	5	6
I expected my provider to care how I feel mentally as well as physically	1	2	3	4	5	6
I expected my provider to be gentle during my physical exam	1	2	3	4	5	6
I expected someone to listen to my problems	1	2	3	4	5	6
I expected a referral when I tell the clinic/office staff about a problem	1	2	3	4	5	6
I expected the services of a social worker to be part of prenatal care	1	2	3	4	5	6
I expected the services of a nutritionist to be part of prenatal care	1	2	3	4	5	6

⇨ GO TO THE NEXT PAGE

YOUR SATISFACTION ABOUT THE PRENATAL CARE RECEIVED IN THE CURRENT PREGNANCY

	I disagree strongly	I disagree moderately	I disagree slightly	I agree slightly	I agree moderately	I agree strongly
I am satisfied with the explanation my provider gave to me of what was going to happen during my prenatal visits	1	2	3	4	5	6
I am satisfied with the explanation my provider gave to me about medical procedures	1	2	3	4	5	6
I am satisfied with the information my provider gave to me about how things are going with my pregnancy	1	2	3	4	5	6
I am satisfied with the kinds of things my provider discussed during my prenatal visits	1	2	3	4	5	6
I am satisfied with the explanation my provider gave to me about what I can expect about parenting a new-born	1	2	3	4	5	6
I am satisfied with the way my provider has prepared me for labour and delivery	1	2	3	4	5	6
I am satisfied with the way my provider treats me	1	2	3	4	5	6
I am satisfied with the respect that I am shown by my provider	1	2	3	4	5	6
I am satisfied with the quality of care that I receive from my provider	1	2	3	4	5	6
I am satisfied with the way I am made to feel that I am not wasting my provider’s time	1	2	3	4	5	6
I am satisfied with being able to ask questions without embarrassment	1	2	3	4	5	6
I am satisfied with not having to repeat my story every time I come in for a visit	1	2	3	4	5	6
I am satisfied with the way the staff expresses concern about my overall personal situation	1	2	3	4	5	6
I am satisfied with the time the staff spends talking about things of interest to me	1	2	3	4	5	6
I am satisfied with the way the staff treats me	1	2	3	4	5	6
I am satisfied with the time the staff takes with me even though I do not have problems with this pregnancy	1	2	3	4	5	6
I am satisfied with the interest and concern the staff has shown me	1	2	3	4	5	6

⇨ GO TO THE NEXT PAGE

	I disagree strongly	I disagree moderately	I disagree slightly	I agree slightly	I agree moderately	I agree strongly
I am satisfied with the way the staff deals with all my medical problems	1	2	3	4	5	6
I am satisfied with the amount of time I wait to be seen by my provider	1	2	3	4	5	6
I am satisfied with the total amount of time I spend at the office/clinic	1	2	3	4	5	6
I am satisfied with the parking facilities of the office/clinic	1	2	3	4	5	6
I am satisfied with the waiting room facilities of the office/clinic	1	2	3	4	5	6
I am satisfied with the examination room of the office/clinic	1	2	3	4	5	6
I am satisfied with my ability to schedule prenatal visits at a time convenient for me	1	2	3	4	5	6
I am satisfied with how easy it is to reschedule my prenatal visits	1	2	3	4	5	6
I am satisfied with how easy it was to get prenatal care early in my pregnancy (that is, before the fourth month)	1	2	3	4	5	6
I am satisfied with having all the recommended tests	1	2	3	4	5	6
I am satisfied with the number of prenatal visits I made until now	1	2	3	4	5	6

If we may contact you in the future for further research please leave your details here:

Tel/GSM:……..

E-mail:……….

May we consult you medical file for further research?

- Yes
- No

If Yes, please fill in your first name and family name:

First Name:……

Family Name:………

Thank you for your contribution to this study.

INSTRUCTIES VRAGENLIJST

Geachte Mevrouw,
Mijn naam is Anna Galle, ik ben vroedvrouw en studente Master in de gezondheidsvoorlichting aan de Universiteit Gent. In het kader van mijn opleiding werk ik aan een thesis over de toegang tot prenatale zorg in Gent. Hiervoor zoek ik zwangere vrouwen die bereid zijn een vragenlijst in te vullen over hun tevredenheid over de prenatale zorg. Het invullen van de vragenlijst duurt ongeveer 10 minuten, ik zal hierbij in de buurt blijven en eventuele vragen beantwoorden. Meer informatie over het onderzoek en het toestemmingsformulier vindt u op pagina 8&9.

De vragenlijst bestaat uit een deel algemene vragen en een deel stellingen. Dit onderzoek staat volledig los van uw zorgverlener van de prenatale consultatie, enkel de onderzoekers zullen inzage krijgen in de door u ingevulde vragenlijst en alles wordt anoniem verwerkt.

VRAGENLIJST: ALGEMENE GEGEVENS

1. Datum waarop je deze vragenlijst invult: ……….. / …………. / 2014

2. Wat is je geboortedatum? ………… / ……….. / 19…………

3. Welke is jouw nationaliteit?

   ……..

4. Welke is de nationaliteit van je ouders?

   Vader: ……………………………………………………………………………………………………….

   Moeder: …………………………………………………………………………………………………….

5. Hoeveel weken ben je momenteel zwanger? ….. weken

6. Jouw hoeveelste zwangerschap is dit (zowel miskramen, abortussen, doodgeboortes en huidige zwangerschap inbegrepen) ?

   ….. zwangerschap

7. Hoeveel levende kinderen heb je nu? ….. kinderen

8. Leeftijd van de kinderen:

   Kind 1: ……………..jaar Kind 2: ……………..jaar

   Kind 3: ……………..jaar Kind 4: ………………jaar

   Kind 5: ……………..jaar Kind 6: ………………jaar

9. Hoeveel miskramen heb je gehad (op minder dan 20 weken zwangerschap)?

   ….. miskramen

10. Was deze zwangerschap gepland?
    - o Ja
    - o Nee

11. Hoeveel weken was je zwanger (in de huidige zwangerschap) toen je voor de eerste keer een prenatale consultatie had bij een vroedvrouw, huisdokter of gynaecoloog?

    ….. weken

12. Hoeveel prenatale consultaties had je tot nu toe bij de vroedvrouw in de huidige zwangerschap?

    ……

13. Hoeveel prenatale consultaties had je tot nu toe bij de gynaecoloog in de huidige zwangerschap?

    ……

14. Hoeveel prenatale consultaties had je tot nu toe bij de huisdokter in de huidige zwangerschap?

……

15. Wat is het hoogste diploma dat je behaalde?
    - o Geen of lager onderwijs
    - o Middelbaar onderwijs
    - o Hoger onderwijs
16. Wat is je huidige gezinssituatie?
    - o Gehuwd of samenwonend
    - o Gescheiden
    - o Latrelatie
    - o Alleenstaand
17. Hoeveel bedraagt het inkomen van jullie gezin?
    - o Minder dan 800 euro
    - o Tussen de 800 en 1000 euro
    - o Tussen de 1000 en 1500 euro
    - o Tussen de 1500 en 2000
    - o Meer dan 2000 euro
18. Indien je een onverwachte rekening van 2824 euro kreeg, hoe eenvoudig zou het voor jou zijn om die binnen de week te betalen?
    - o Geen probleem
    - o Een beetje moeilijk
    - o Heel moeilijk
19. Heb je ooit gerookt?
    - o Nee, nooit gerookt
    - o Ja
20. Rook je nu?
    - o Ja, minstens 1 sigaret per dag
    - o Ja, zo nu en dan (minstens 1 sigaret per week, niet dagelijks)
    - o Zelden (minder dan 1 sigaret per week)
    - o Neen

GA DOOR NAAR DE VOLGENDE PAGINA =>

21. Hoe vaak drink je alcohol?
    - Nooit
    - Niet tijdens de zwangerschap
    - Occasioneel, minder dan 1 keer per maand
    - één tot drie keer per maand
    - één of twee keer per week
    - (bijna) alle dagen
22. Heb je ooit slaappillen, kalmeermiddelen, angstremmers, antidepressiva of andere medicijnen voor psychische problemen gebruikt?
    - Nooit
    - Ja, maar niet in de 12 maanden voorafgaand aan de zwangerschap
    - Ja, in de 12 maanden voorafgaand aan de zwangerschap
    - Ja, tijdens de zwangerschap
23. Heb je ooit drugs zoals cannabis, amfetamines, extasy of cocaïne gebruikt ?
    - Nooit
    - Ja, maar niet in de 12 maanden voorafgaand aan de zwangerschap
    - Ja, in de 12 maanden voorafgaand aan de zwangerschap
    - Ja, tijdens de zwangerschap
24. Heeft je (ex-)partner je met de vuist geslagen, met de vlakke hand geslagen, geschopt of op een andere manier fysiek pijn gedaan?
    - Ja, in de 12 maanden voorafgaand aan de huidige zwangerschap
    - Ja, in de huidige zwangerschap
    - Nee
25. Heeft je (ex-)partner je op een seksuele manier aangeraakt wanneer je dat niet wou of je gedwongen tot seksuele activiteiten?
    - Ja, in de 12 maanden voorafgaand aan de huidige zwangerschap
    - Ja, in de huidige zwangerschap
    - Nee
26. Heeft je (ex-)partner je vernederd of gekleineerd ten opzichte van andere mensen, dingen gedaan om je opzettelijk bang te maken, je opzettelijk geïntimideerd of op een andere manier emotioneel misbruikt?
    - Ja, in de 12 maanden voorafgaand aan de huidige zwangerschap
    - Ja, in de huidige zwangerschap
    - Nee

We vragen je de stellingen te beoordelen op een schaal van 1 tot 6, van helemaal niet akkoord tot helemaal akkoord. Probeer je te herinneren welke verwachtingen je had aan het begin van de zwangerschap over de prenatale zorg en op basis daarvan de stellingen te beoordelen.

JOUW VERWACHTINGEN AAN HET BEGIN VAN DE ZWANGERSCHAP

	Helemaal niet akkoord	Niet akkoord	Eerder niet akkoord	Eerder akkoord	Akkoord	Helemaal akkoord
Ik verwachtte sneller een afspraak te krijgen voor mijn eerste prenatale consultatie	1	2	3	4	5	6
Ik verwachtte dat mijn prenatale consultaties lang zouden duren	1	2	3	4	5	6
Ik verwachtte dat de prenatale consultatie meer zou inhouden dan de controle van mijn gewicht en de hartslag van mijn baby	1	2	3	4	5	6
Ik verwachtte dat ik informatie zou krijgen tijdens de prenatale consultatie zonder dat ik zoveel vragen moest stellen	1	2	3	4	5	6
Ik verwachtte één zorgverlener (=vroedvrouw, gynaecoloog of dokter) voor de prenatale consultaties die ik regelmatig zou zien	1	2	3	4	5	6
Ik verwachtte dat diezelfde zorgverlener ook mijn bevalling zou doen	1	2	3	4	5	6
Ik verwachtte dat mijn zorgverlener zowel voor mijn mentaal als fysiek welzijn zou zorgen	1	2	3	4	5	6
Ik verwachtte dat mijn zorgverlener bij een lichamelijk onderzoek voorzichtig en zacht zou zijn	1	2	3	4	5	6
Ik verwachtte iemand die naar mijn problemen zou luisteren	1	2	3	4	5	6
Ik verwachtte doorverwezen te worden wanneer ik aan iemand van het onthaal of een zorgverlener over een probleem zou vertellen	1	2	3	4	5	6
Ik verwachtte de dienstverlening van een sociaal assistent als onderdeel van de prenatale zorg	1	2	3	4	5	6
Ik verwachtte de dienstverlening van een diëtist als onderdeel van de prenatale zorg	1	2	3	4	5	6

⇨ GA DOOR NAAR DE VOLGENDE PAGINA

JOUW TEVREDENHEID OVER DE PRENATALE ZORG IN DE HUIDIGE ZWANGERSCHAP

	Helemaal niet akkoord	Niet akkoord	Eerder niet akkoord	Eerder akkoord	Akkoord	Helemaal akkoord
Ik ben tevreden over de uitleg van mijn zorgverlener over wat er tijdens de prenatale consultaties zou gebeuren	1	2	3	4	5	6
Ik ben tevreden over de uitleg van mijn zorgverlener over medische procedures	1	2	3	4	5	6
Ik ben tevreden over de informatie die mijn zorgverlener gaf over hoe het gaat met mijn zwangerschap	1	2	3	4	5	6
Ik ben tevreden over de dingen die mijn zorgverlener besprak tijdens mijn prenatale controles	1	2	3	4	5	6
Ik ben tevreden over de uitleg die mijn zorgverlener gaf over wat ik kan verwachten van het ouderschap	1	2	3	4	5	6
Ik ben tevreden over de manier waarop mijn zorgverlener mij heeft voorbereid op de arbeid en de bevalling	1	2	3	4	5	6
Ik ben tevreden over de manier waarop mijn zorgverlener mij behandelt	1	2	3	4	5	6
Ik ben tevreden over het respect dat de zorgverlener heeft voor mij	1	2	3	4	5	6
Ik ben tevreden over de kwaliteit van de zorgverlening	1	2	3	4	5	6
Ik ben tevreden over de manier waarop mijn zorgverlener laat aanvoelen dat ik niet zijn/haar tijd aan het verspillen ben	1	2	3	4	5	6
Ik ben tevreden dat ik zonder schaamte vragen kan stellen	1	2	3	4	5	6
Ik ben tevreden dat ik niet telkens opnieuw mijn verhaal moet vertellen wanneer ik op consultatie kom	1	2	3	4	5	6
Ik ben tevreden over de manier waarop het medisch personeel betrokkenheid toont bij mijn persoonlijke situatie	1	2	3	4	5	6
Ik ben tevreden met de tijd die men spendeert aan het bespreken van zaken die belangrijk zijn voor mij	1	2	3	4	5	6
Ik ben tevreden over de manier waarop het medisch personeel mij behandelt	1	2	3	4	5	6
Ik ben tevreden met de tijd die men spendeert aan mij, ook al heb ik geen problemen in de huidige zwangerschap	1	2	3	4	5	6
Ik ben tevreden over de interesse en bezorgdheid die wordt getoond door het medisch personeel	1	2	3	4	5	6

	Helemaal niet akkoord	Niet akkoord	Eerder niet akkoord	Eerder akkoord	Akkoord	Helemaal akkoord
Ik ben tevreden over de manier waarop het medisch personeel omgaat met mijn medische problemen	1	2	3	4	5	6
Ik ben tevreden over de wachttijden voor de consultatie	1	2	3	4	5	6
Ik ben tevreden over de totale tijd die ik doorbreng in het ziekenhuis /de privépraktijk	1	2	3	4	5	6
Ik ben tevreden over de parkeermogelijkheden van het ziekenhuis/de privépraktijk	1	2	3	4	5	6
Ik ben tevreden over de wachtkamers van het ziekenhuis/de privépraktijk	1	2	3	4	5	6
Ik ben tevreden over de onderzoekskamer van het ziekenhuis/de privépraktijk	1	2	3	4	5	6
Ik ben tevreden dat ik mijn prenatale consultatie kan plannen op een moment dat het mij past	1	2	3	4	5	6
Ik ben tevreden over de flexibiliteit waarmee prenatale consultaties kunnen worden verzet	1	2	3	4	5	6
Ik ben tevreden dat het zo gemakkelijk was om vroeg in mijn zwangerschap (voor de vierde maand) prenatale zorg te krijgen	1	2	3	4	5	6
Ik ben tevreden dat ik alle aanbevolen onderzoeken en testen krijg	1	2	3	4	5	6
Ik ben tevreden over het aantal prenatale consultaties tot nu toe	1	2	3	4	5	6

Indien we je in de toekomst voor bijkomend onderzoek mogen contacteren, mag je hier je gegevens achterlaten:

Tel/GSM:……….

E-mail:………

Mogen we jouw medisch dossier raadplegen voor verder onderzoek?

- Ja
- Nee

Indien ja, gelieve dan jouw voornaam en familienaam op te schrijven:

Voornaam:……

Familienaam:………

Hartelijk dank voor je bijdrage aan dit onderzoek.
